# Toe brachial index and not ankle brachial index is appropriate in initial evaluation of peripheral arterial disease in type 2 diabetes

**DOI:** 10.1186/s13098-024-01291-2

**Published:** 2024-02-27

**Authors:** Pankaj Singhania, Tapas Chandra Das, Chiranjit Bose, Asif Mondal, Rana Bhattacharjee, Archana Singh, Satinath Mukhopadhyay, Subhankar Chowdhury

**Affiliations:** 1 Department of Endocrinology and Metabolism, Institute of Post Graduate Medical Education and Research, Kolkata, India; 2 Department of Radiodiagnosis, Institute of Post Graduate Medical Education and Research, Kolkata, India; 3 Department of Endocrinology, Medical College, Kolkata, India

**Keywords:** Type 2 diabetes, Peripheral arterial disease, Toe brachial index, Ankle brachial index, CT angiography

## Abstract

**Background:**

Non-invasive clinic-based tools for assessing PAD are not without limitations. Therefore, costly tests like Doppler study, CT angiography and MR angiography are often required to make a diagnosis. Ankle brachial index (ABI), commonly used for assessment of PAD, has high false positivity rates in sclerosed, calcified arteries which render them non-compressible. Toe brachial index (TBI) can be an alternative, as digital arteries are relatively unaffected by these changes.

**Aim:**

To compare the reliability of ABI and TBI in diagnosing PAD in type 2 diabetes using CT angiography (CTA) as the reference.

**Methods:**

175 adults with T2D were selected. ABI &TBI were measured with an automated vascular Doppler XT 6 ports bilaterally for all subjects. For any subject, the limb with lower ABI and TBI was included for analysis. ABI < 0.9 & TBI < 0.6 were taken as evidence of PAD. CTA showing > 50% narrowing was taken as evidence of PAD.

**Results:**

24% of our study subjects had CTA confirmed PAD. ABI has low sensitivity of 35.29% (95% CI 0.21–0.52) compared to TBI being 82.35% (95% CI 0.66–0.92). The specificity however was similar. ABI < 0.9 was able to detect CTA confirmed PAD, but ABI > 0.9, including the so-called normal ABI (0.9–1.3) was unable to detect PAD. ROC showed ABI at 1.005 has sensitivity 64.71% (95% CI 0.48- 0.79) and specificity 61.7% (95% CI 0.53–0.69) and TBI at 0.6 has sensitivity 82.35% (95% CI 0.66–0.92) & specificity 92% (95% CI 0.87–0.96). Utilizing Cohen’s Kappa, the reliability of ABI with respect to CTA showed fair agreement (K = 0.225, p = 0.001), whereas the reliability of TBI with respect to CTA showed substantial agreement (K = 0.759, p < 0.0001).

**Conclusion:**

ABI < 0.9 detects PAD reliably, but presence of PAD in patients with ABI > 9.0 including the normal of ABI (0.9–1.3) can be confirmed with TBI, which correlated strongly with CTA. TBI is also non-inferior for PAD detection, when ABI < 0.9. TBI and not ABI can be utilized for initial assessment of PAD in subjects with T2D.

**Graphical Abstract:**

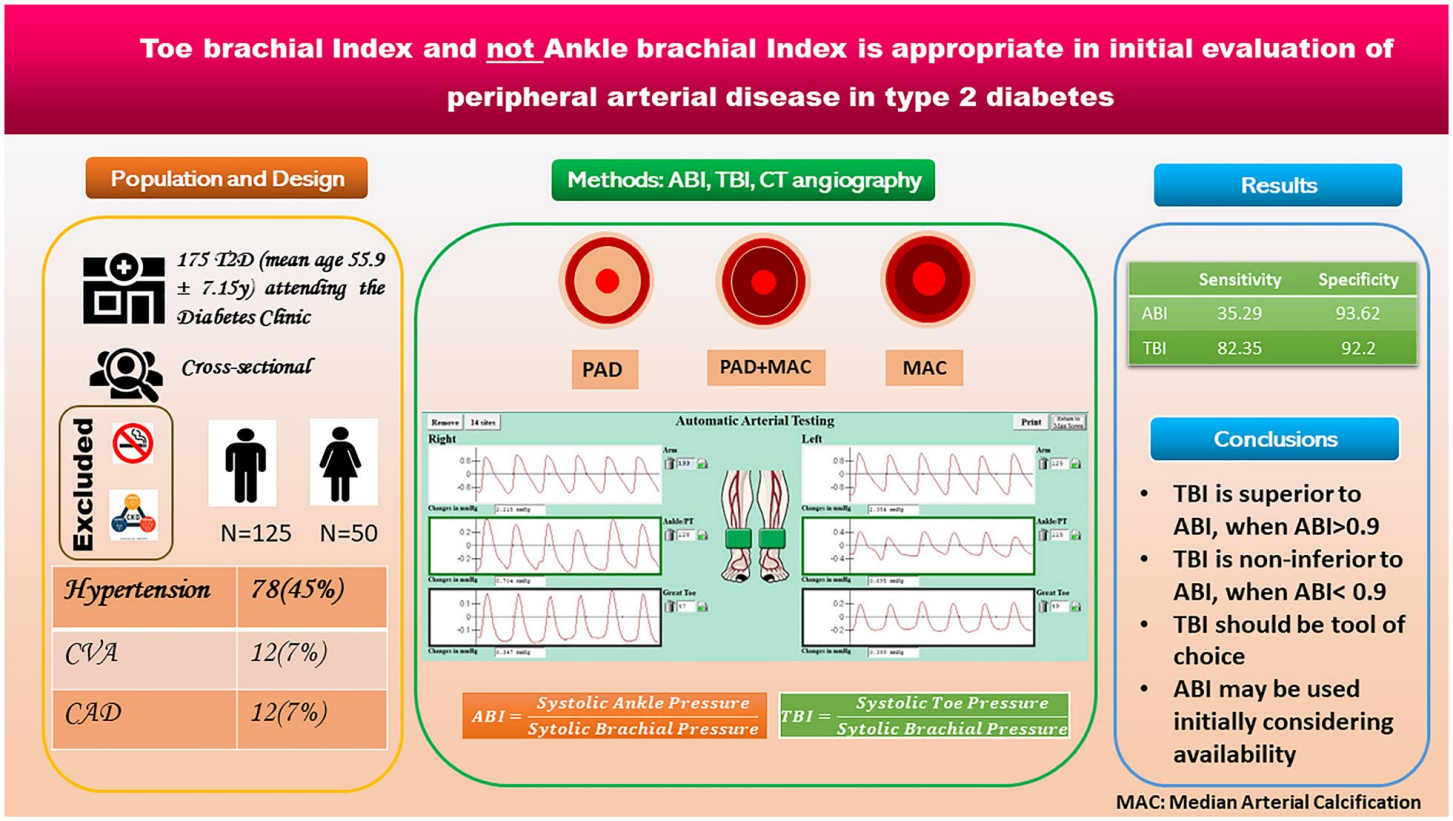

**Supplementary Information:**

The online version contains supplementary material available at 10.1186/s13098-024-01291-2.

## Background

Peripheral arterial disease (PAD) is characterized by atherosclerotic occlusive disease of the lower extremities and is a marker of atherothrombotic disease in other vascular beds. Therefore, patients with PAD are at an increased risk of stroke, myocardial infarction, and death. PAD is also a major risk factor for lower extremity ulceration, non-healing of ulcers and amputation in people with diabetes [1]. Diabetes and smoking are the strongest risk factors for PAD. Other established risk factors are advanced age, hypertension, and dyslipidemia. [2].

Ankle brachial index (ABI) is a time tested and reliable tool, often the first test used for PAD screening in subjects with type 2 diabetes [3, 4]. However, recent research suggests that the diagnostic accuracy of ABI is compromised in certain subsets of population. Decreased sensitivity and specificity of ABI for the presence of PAD have been seen in various studies in the elderly and in those with chronic kidney disease or diabetes [5, 6]. There is now enough evidence to prove that higher rates of medial arterial calcification (MAC) in these specific populations leads to arterial wall stiffening, thus preventing full compression of the lower extremity arteries during vascular testing. This results in falsely elevated ABI, reducing the usefulness of the test [7]. While ABI > 1.3 has been recognized as a marker of vascular calcification, the performance of ABI < 1.3 needs to be explored, particularly in high-risk population like diabetes where arterial calcification is widespread, subclinical and may affect ABI measurement [8].

An alternative method of non-invasive vascular assessment using small vessel–testing is the toe-brachial index (TBI). Toe arteries are unaffected by vascular calcification due to their narrow caliber. Therefore, toe pressure or TBI, can be a useful alternative to ABI [9–14]. However, the utility of TBI as a vascular assessment tool is largely underrecognized due to unavailability and lack of data regarding the accuracy of TBI and ABI in diagnosing PAD using diagnostic imaging modalities as reference standard.

The aim of our study was to determine the diagnostic accuracy of ABI and TBI for PAD in subjects with T2D.

## Material and methods

### Study design and population

This study was undertaken at the department of Endocrinology and metabolism, Diabetes outpatient department (OPD), Institute of Post Graduate Medical Education & Research, Kolkata, India. Ethical approval was obtained from the institutional ethics committee. Informed written consent were taken before participation. For this cross-sectional study, we recruited 175 subjects with type 2 diabetes (T2D) attending our diabetes OPD from April 2021–June 2022. Participants were selected based on lower extremity vascular screening criteria [15].

The inclusion criteria were any subject with T2D more than 50-year-old. Current or ex-smokers, subjects with contraindications to ankle or toe pressure measurement, like those with active great toe infection or ulceration, absent great toe, vasospastic disorders, cardiac arrhythmias, and history of deep vein thrombosis or lymphedema were excluded from the study. Subjects with CKD as determined eGFR < 60 ml/min/1.73m2 were also excluded.

### Sample size calculation

Sample size for our study was calculated based on Fisher’s formula:

$${\text{N}}=\frac{{{\text{Z}}}^{2}{\text{Pq}}}{{{\text{d}}}^{2}}$$

where N = minimum sample size, Z = the standard normal deviate corresponding to a 95% confidence level (1.96). P = prevalence of peripheral artery disease in subjects with T2DM that is 6.3% [23], Q = complementary probability i.e., 1 − P = 1 − 0.063 = 0.937, and d = absolute precision limit desired (5%) = 0.05. Thus N = (1.96)2 (0.063) (0.937)/ (0.05)2 ≈ 91. Subjects were recruited on each diabetes out-patient clinic day until a sample size of 91 was attained. Beyond that we continued to recruit subjects and at the time of manuscript writing, we had recruited 175 subjects.

### Clinical examination and baseline data collection

Anthropometry for all subjects were recorded. Height was measured with a wall mounted stadiometer; weight was recorded with a Essae DS 415 weighing scale. Both height and weight were rounded off to the first decibel. Body mass Index (BMI) was calculated using the formula weight (kg)/Height (m) ^2^. Blood pressure (systolic, SBP and diastolic, DBP) were recorded in the left arm in sitting posture after 15 min rest for all subjects with a manual aneroid instrument. Average of three readings were taken to arrive at the final value. Blood samples for serum creatinine, lipid profile, fasting glucose and glycated hemoglobin were collected after 10–12 h overnight fasting. Serum creatinine was estimated by modified Jaffe’s method. Serum triglyceride was estimated by glycerol peroxidase method. Serum LDL and HDL were measured by assay based on polyvinyl sulfonic acid (PVS) polyethylene glycol methyl ether (PEGME) coupled classic precipitation method. Fasting plasma glucose was measured by glucose oxidase peroxidase method. These blood tests were measured on a fully automatic Erba diagnostics equipment, Mannheim, Germany. High performance liquid chromatography (HPLC) was used to estimate glycated hemoglobin (HbA1c) on Biorad 10.

### ABI and TBI estimation

All participants were subjected to non-invasive vascular testing for recording ABI and TBI bilaterally. The recordings were performed by one of two investigators. During the testing session, participants were allowed to rest for 10 min in supine position before the recording was done. Temperature of the testing room was maintained between 23 and 25 °C by air-conditioning. We used the fully automatic Hadeco XT -6 ports (Hadeco Inc Japan) for recording ABI and TBI. Six cuffs each designated for the arm, ankle and toe of each side were applied simultaneously. Brachial and ankle pressure were recorded by oscillometry and toe pressure by photoplethysmography (PPG) with the help of a sensor applied to the distal pulp of each great toe fixed with Velcro. While taking toe pressure with PPG probe, the unit senses the refection of light from the hemoglobin of the red blood cells in the surface vessels by utilizing infrared light with the probe. The Doppler instrument gave the calculated ABI and TBI bilaterally on a computer software-based interface. For uniformity the posterior tibial artery was used for ankle pressure bilaterally in all subjects. Lower of the two readings (left and right leg) was taken as the final ABI and TBI for that subject. ABI less than 0.9 was taken as evidence of PAD. This has been the standard cut-off for ABI across previous studies [16]. Similarly, TBI less than 0.6 was taken as evidence of PAD [17]. The different cut-offs for ABI and TBI originate from the fact that normal toe pressure is lower compared to ankle pressure because of the narrow caliber of toe arteries. 10 random subjects were asked to come after 1 week and vascular assessment (ABI, TBI) was repeated by second investigator who was blinded to the initial findings, to determine inter-rater reliability.

### CT angiography

CTA of both lower limb arterial system was performed with a SIEMENS 128 slice, Somatom definition, single source CT scanner. Both lower limbs vessels were studied form the abdominal aorta down to the distal ankle. The lowest possible radiation and contrast exposure was used. Presence of stenosis of one or more artery of > 50% on any side was taken as evidence of PAD. CTA was used as the non-invasive gold standard for our study. The sensitivity, specificity, positive predictive value (PPV), negative predictive value (NPV) and likelihood ratio of ABI and TBI were calculated against CTA taking an ABI cut-off of < 0.9 and a TBI cut-off of < 0.6 for PAD detection.

### Statistical analysis

D'Agostino & Pearson test was performed to check the normality of the distribution of variables. Descriptive continuous variables were represented by Mean and SD (normally distributed variables) or median and interquartile range (non-normally distributed variables). Unpaired t test and Mann Whitney test was performed between two groups. Chi-squared test was performed for categorical data. Fisher's exact test was used where a specific group contains < 7 subjects. P value less than 0.05 was considered as statistically significant. For ROC analysis, Wilson/Brown test was performed and CI was taken at 95%. ROC was plotted for ABI and TBI. Pearson’s correlation test and Spearman’s correlation test was used to test correlation statistics in the normally and non-normally distributed parameters. Correlation was determined between ABI and TBI across all ranges of ABI. ABI readings were converted to dichotomous variable using 0.9 as PAD cut-off. Similarly, TBI was also converted to dichotomous variable using 0.6 as PAD cut-off. Thereafter, the reliability of both ABI and TBI were determined with respect to CTA according to presence or absence of PAD as a dichotomous variable using unweighted Cohens K statistics. Intraclass correlation coefficients (ICCs) with 95% confidence interval (CI) were calculated to determine the level of agreement between test and retest for the ABI and TBI. Cut-offs suggested by Fleiss were used to interpret the ICC values for intertester reliability [18] Cohen’s kappa statistic was interpreted by the method proposed by Landis and Koch [19]. Statistical analysis was performed by using GraphPad Prism Software version 9.0 and SPSS version 26. All values were rounded off to one decimal.

## Results

### Baseline characteristics

Based on CTA, the study population was divided into two arms: PAD positive and PAD negative. 42/175 (24%) of the study subjects were found to have CTA confirmed PAD. 71.4% subjects were male Table 1 shows the chief features including demographic features of the study population. Other features were not significantly different in the two groups.

**Table 1:** Demographic and baseline characteristics of the study subjects

Variables		PAD positive (n = 42)	PAD negative (n = 133)	p value
Age (years)		59.1 ± 7.3	55.1 ± 7.0	**0.0010**
Gender	Male	37 (88.1)	88 (66.3)	**0.0226**
	Female	5 (11.9)	45 (33.8)	
Past stroke	Yes	3 (7.1)	9 (6.8)	0.790
	No	39 (92.9)	124 (93.2)	
Past CAD	Yes	6 (14.3)	6 (4.5)	0.0665
	No	36 (85.7)	127 (95.5)	
H/o Claudication	Yes	27 (62)	13 (9.7)	**0.0016**
	No	15(38)	120 (91.3)	
Duration of Diabetes (years)		13.0 ± 5.4	13.45 ± 5.2	0.3702
Body mass index (BMI) Kg/m^2^		24.2 ± 3.7	24.9 ± 3.4	0.1455
Hypertension (> 140/90 mm Hg)	Yes	19 (45.2)	59 (44.4)	0.3050
	No	23 (54.8)	74 (55.6)	
Serum triglyceride(mg/dl)		134.0 ± 56.6	169.5 ± 85.9	0.0061
Serum LDL (mg/dl)		90.5 ± 26.7	101.3 ± 34.1	0.0784
Serum HDL mg/dl)		40.39 ± 8.8	41.20 ± 8.7	0.5764
Fasting plasma glucose (mg/dl)		142.5 ± 56.8	141.1 ± 59.4	0.8659
Glycated hemoglobin (HbA1c) g%		8.9 ± 2.3	8.4 ± 1.9	0.3655

Data are in n (%) or mean ± SD

PAD positive and PAD negative: CTA based diagnosis; ABI: ankle brachial index, TBI: Toe brachial index, LDL: low density lipoprotein, HDL: high density lipoprotein

**Fig. 1:**
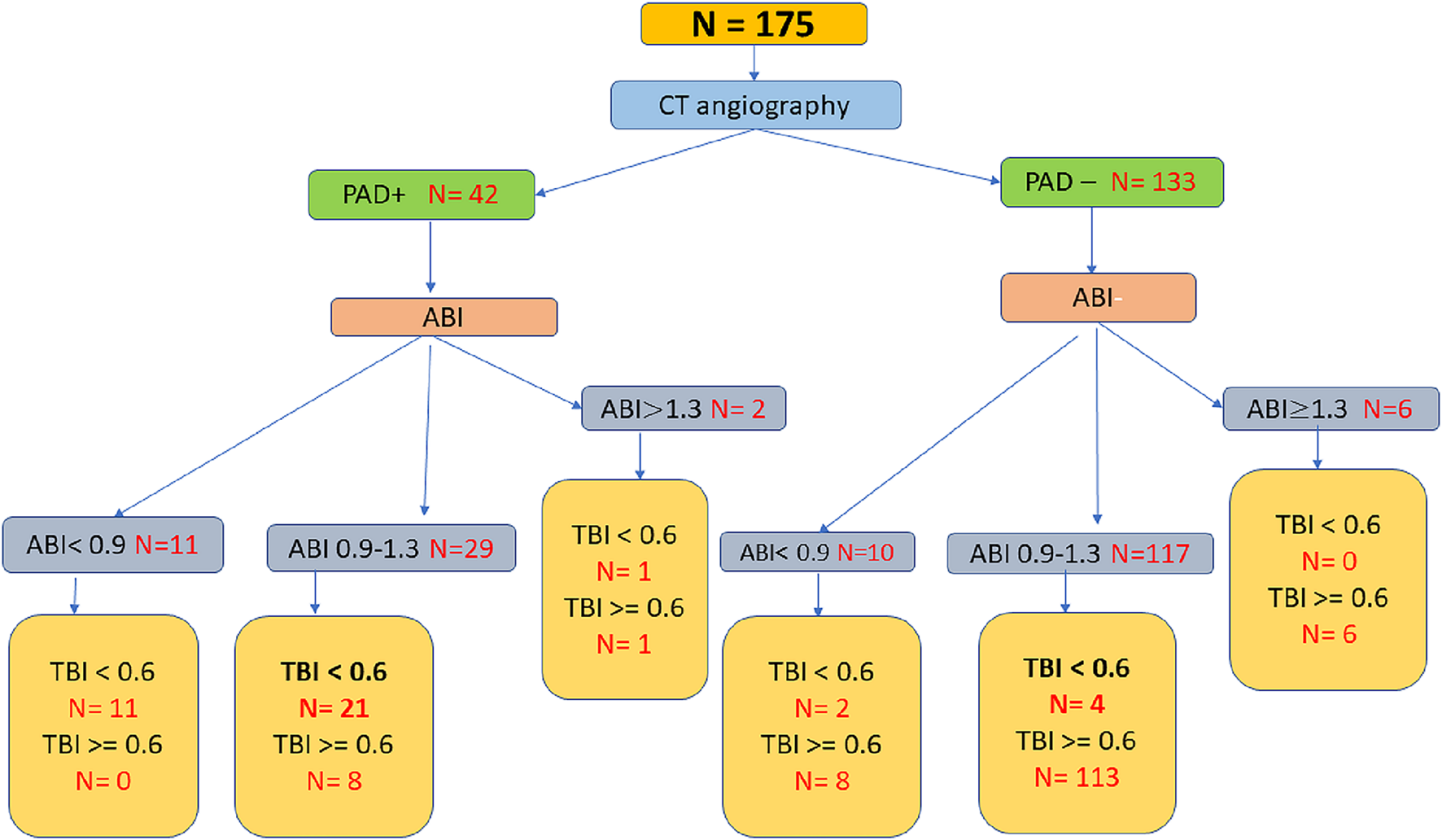
Figure 1 shows the major findings of the study. The flow chart shows the study pathway in details with major findings. Starting with 175 subjects the chart shows the findings based on CTA, ABI and TBI for all subjects. The most striking areas of interest are the segment showing the normal ABI (0.9–1.3), where we see a large chunk of subjects have CTA proven PAD and a low TBI.TBI is non-inferior in people with ABI < 0.9 and is superior in people with ABI > 0.9

On careful examination, Table 1 we can draw some conclusions. The average age of subjects with peripheral arterial disease is higher than those without PAD and the difference is also statistically significant. Males have more PAD compared to females. History of claudication was reported more among those with CTA detected PAD (Fig. 1).

### Diagnostic performance

Table 2 is the heart of our study. A careful look at the data shows the diagnostic performance of ABI and TBI when compared to CTA. While ABI and TBI have high and almost equal specificity at diagnosing PAD in T2D, the sensitivity of ABI is quite low compared to TBI. This essentially means that a normal ABI is likely to miss a diagnosis of confirmed PAD. TBI on the other hand does not miss confirmed PAD cases. Both ABI and TBI are equally able to exclude those without PAD. The Likelihood ratio for the two testing modalities also reflects similar trend.

**Table 2:** Sensitivity, specificity, positive predictive value, negative predictive value and likelihood ratio of the ankle brachial index and toe brachial index compared with CT angiography

	Sensitivity (95% CI) %	Specificity (95%CI) %	NPV (95% CI) %	PPV (95% CI) %	Likelihood ratio (LR)
ABI	35.29 (0.21 to 0.52)	93.62 (0.88 to 0.97)	57.14 (0.37 to 0.76)	85.71 (0.79 to 0.90)	5.53
TBI	82.35 (0.66 to 0.92)	92.20 (0.87 to 0.96)	71.79 (0.56 to 0.83)	95.59 (0.91 to 0.98)	10.56

### Correlation

**Fig. 2:**
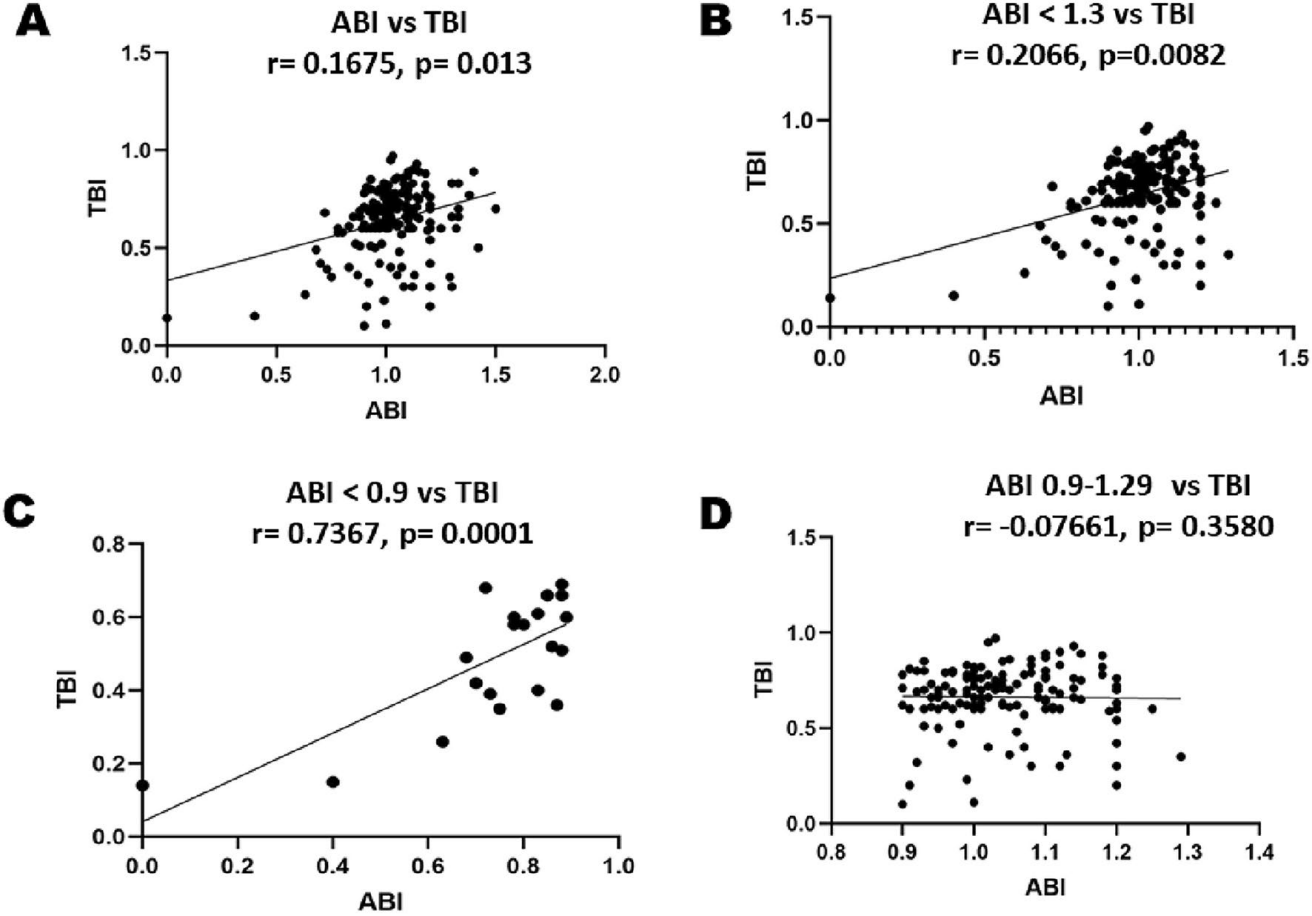
**A** Correlation plot between ABI and TBI showing positive correlation with statistical significance, though the correlation is weakly positive. **B** Correlation plot between ABI < 1.3 and TBI showing positive correlation with statistical significance. **C** Correlation plot between ABI < 0.9 and TBI showing strong positive correlation with statistical significance. **D** Correlation plot between ABI 0.9–1.3 and TBI showing no correlation between the two variables

Additional file 1: Table S1 and Fig. 2A–D give detailed values of the correlation between ABI and TBI. It is evident from the table that there is good positive correlation between ABI and TBI when the ABI is less than 0.9. Whereas for an ABI more than 1.3 the correlation with TBI is very poor. Interestingly the correlation of a so-called normal. ABI (0.9–1.3) with TBI is even poor, or rather negative.

### ROC

**Fig. 3:**
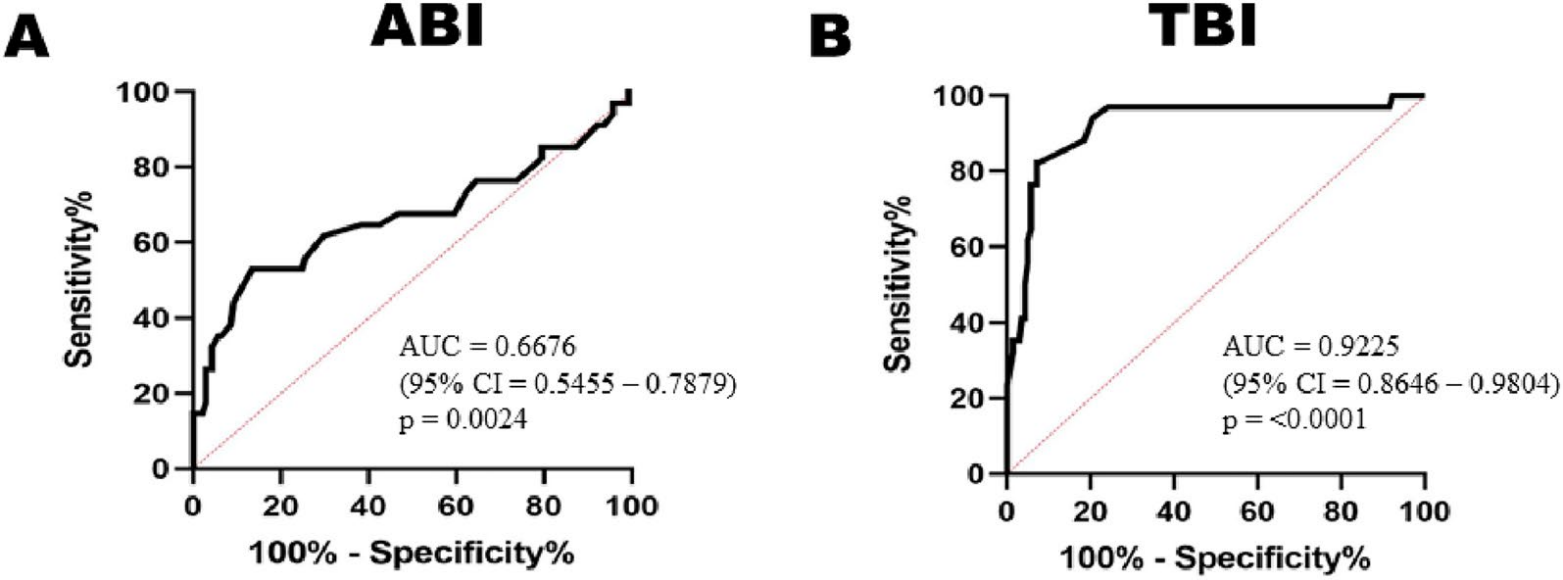
Receiver operating curve of ABI (**A**) and TBI (**B**)

Receiver operating curves (ROC) for ABI and TBI are depicted in Fig. 3A and B respectively. Area under the curve (AUC) for ABI is 0.67 (95% CI 0.54–0.79) and the analysis was statistically significant (p = 0.002). AUC for TBI was 0.92 (95% CI 0.86–0.98) with a p value < 0.0001. The area under curve (AUC) is higher for TBI compared to ABI. Using the ROC analysis, we also decided to find out the best tradeoff value for ABI and TBI. ROC showed, ABI of < 1.005 has a 64.71% sensitivity and 61.7% specificity for PAD detection whereas, TBI < 0.595 has an 82.35% sensitivity and 92.2% specificity for PAD detection.

Utilizing Cohen’s Kappa, the reliability of ABI with respect to CTA showed fair agreement (K = 0.225, p = 0.001), whereas the reliability of TBI with respect to CTA showed substantial agreement (K = 0.759, p < 0.0001) [17]. The ICCs showed excellent test–retest reliability for both ABI (ICC, 0.990; 95% CI, 0.960–0.998) and TBI (ICC, 0.983; 95% CI, 0.931–0.996) [18].

## Discussion

Ankle brachial Index is commonly used as an office tool to detect PAD in subjects with T2D, but has low sensitivity. This limitation can be overcome with toe brachial index and it should be the initial tool to assess PAD in these subjects. This will help detect PAD early and prevent long term complications.

Peripheral arterial disease is a common yet underrated long term complication of type 2 diabetes. PAD is a major cause of lower limb ulceration, non-healing of foot ulcers, amputation, and mortality in people with diabetes [20]. Although much emphasis has been on peripheral neuropathy as a foot complication in diabetes, PAD is an equal contributor [21].

The prevalence of PAD is a matter of constant debate and there are various estimates worldwide. It has largely been believed that PAD is less common in India compared to the Caucasian population [22]. The data from India is also highly heterogeneous. A study from Eastern India based on ABI, gave a very high PAD prevalence of 36% in subjects with T2D [23]. Another study from north India showed a very low prevalence of PAD in T2D at 1.49% [24]. A study from Kerala, India estimated a prevalence of 26% and that from Chennai, India 6.3% [25]. Using this prevalence (6.3%), based on CTA, we arrived at a PAD prevalence of 24% in people with T2D. It is thus evident that prevalence of PAD in T2D varies from one part of the country to another and largely depends on the diagnostic tool used and cut-offs decided upon. Nonetheless, PAD in T2D is not as rare in India as was previously thought and we have ample evidence including the present study to substantiate this.

While diabetes is associated with PAD, there are some established risk factors which increase PAD. The basic pathophysiology of PAD is atherosclerosis and this increases with age. Therefore, increasing age is an accepted risk factor for PAD [26]. We also found in our study that subjects with PAD were older compared to those without PAD. To further elucidate the effect of age as a confounder, correlation between age and ABI / TBI was determined. There was no correlation between age and either ABI or TBI. PAD has traditionally been considered to be more common in men. Though recent studies have shown that women are equally affected especially after menopause [27]. In our population we found males had more CTA confirmed PAD compared to women. Claudication is the development of pain deep in the muscles of the leg, relieved quickly with rest. Claudication is suggestive of PAD but is a non-specific sign and may be absent in PAD due to concomitant neuropathy. Moreover, diabetics without PAD may complain of calf pain [28, 29]. Claudication was reported in a substantial number of our subjects with PAD, but we do not attribute much importance to this and rely on diagnostic tools for PAD. Other traditional risk factors like dyslipidemia and hypertension were not found to be different in those with PAD compared to those without PAD. The presence of these risk factors would not affect our findings as these are traditional risk factors. These will act as contributors rather than confounders.

Catheter based angiography remains the gold standard for diagnosing PAD, but it is invasive and cannot be offered as an OPD screening tool [30]. Therefore, non-invasive OPD based tools have been used for the screening high risk subjects for PAD including those with diabetes. The available non-invasive tests can be simple OPD tools like ABI, TBI and TcPO2 or laboratory-based modalities like USG color Doppler, CTA, and MR angiography (MRA). ABI has been the most used technique to screen for PAD. ABI is simple, cheap, readily available, easy to obtain and has good sensitivity in healthy individuals [31–34]. However it is plagued with a poor sensitivity to diagnose PAD in high-risk subjects like people with diabetes and renal disease [35]. We also found in this study that ABI has a poor sensitivity of about 35% for diagnosing PAD in subjects with diabetes. This means ABI will miss subjects with confirmed PAD. The specificity on the other hand is an impressive 93% meaning that ABI will not diagnose PAD falsely in those without the disease. The poor performance of ABI as a screening tool is largely due to medial arterial calcification in high-risk subjects especially those with diabetes. MAC renders peripheral arteries non-compressible and falsely elevates ankle pressure and ABI [36].

It has been reported that ABI > 1.3 is associated with non-compressible arteries and ABI > 1.3 is considered falsely elevated and can miss a diagnosis of PAD. Since diabetes is associated with early and widespread arterial calcification, the so-called normal ABI (ABI 0.9–1.3) can also be affected by MAC and may miss PAD [37]. In our study we found that among those with CTA confirmed PAD, about 65% subjects had normal or raised ABI. The number of subjects with normal ABI (0.9–1.3) was very high and had we relied upon ABI as the only tool for PAD screening many subjects would have been wrongly declared as not having PAD.

Here comes the need for an alternative tool for PAD screening. Toe arteries are small caliber arteries and not affected by wall calcification. This would mean that the problem of non-compressible arteries would not apply to toe arteries and that toe pressure would not be falsely elevated. TBI calculated using toe pressure and brachial pressure can come to our rescue when ABI is not useful [38]. We found that TBI has a sensitivity of more than 82% in diagnosing PAD in subjects with diabetes. The specificity of TBI was also impressive and like ABI. Thus, TBI was less likely to either miss CTA confirmed PAD or falsely diagnose PAD in those without the disease. Reliability testing also demonstrated substantial agreement with CTA diagnosed PAD of TBI compared to ABI. Therefore, TBI was a better tool for PAD detection and may substitute and not just complement ABI. The correlation between ABI and TBI was also determined as part of the present study. We found that ABI < 0.9 had a good positive correlation with TBI, so TBI is non-inferior in people with ABI < 0.9. On the contrary, ABI > 1.3 as well as so-called normal ABI (0.9–1.3) had very poor correlation with TBI. The only drawback with TBI is cost and lack of widespread availability of the equipment. Current recommendations regarding use of TBI are largely restricted to ABI > 1.3, but this does not consider the fact that medial arterial calcification in PAD may affect ABI even within the normal range [39–41]. We conclusively prove from our data that the ABI 0.9–1.3 may not be normal and thus miss the diagnosis of PAD. All subjects with ABI > 0.9 (normal and elevated) should be subjected to TBI recordings to diagnose PAD. For those with ABI < 0.9 there was good agreement with TBI values and CTA findings. Therefore, in this category (ABI < 0.9) the findings can be taken as evidence of PAD and need no further screening. Receiver operating curve (ROC) analysis for ABI and TBI were also obtained in the present study. The area under the curve (AUC) for TBI was higher than ABI. The ROC analysis was also used to obtain a best trade -off value for ABI and TBI. We found that an ABI of < 1.005 has a 64.71% sensitivity and 61.7% specificity for PAD detection whereas, a TBI < 0.6 has 82.35% sensitivity and 92.2% specificity for PAD detection. Best tradeoff for TBI is approximately 0.6 which is the cut-off currently accepted as diagnostic of PAD. TBI > 0.6 is believed to exclude PAD. ROC showed ABI < 1.0 to be a better cut-off for diagnosis of PAD. Compared to CTA and TBI, ABI < 1.0 (instead to 0.9) had better sensitivity and superior NPV for detection of PAD, however the specificity and PPV was lower. Therefore, altering the ABI cut-off may offer some advantage in terms of detecting those with PAD, the overall performance can be improved only if it is complemented with TBI.

CTA is an excellent modality for PAD diagnosis. The sensitivity and specificity of multidetector CTA compared with catheter based invasive angiography is ≈90% for detecting PAD [42]. We performed CTA in our study subjects with the lowest possible radiation and contrast dose. Subjects with CKD were also excluded from the study. USG color Doppler was also conducted on all our study subjects. Though USG color Doppler is highly operator dependent and believed to be less sensitive, we found good correlation between CTA and USG color Doppler in our study subjects with T2D. Therefore, CTA can be avoided in subjects with suspicion of PAD unless revascularization is contemplated. This will avoid cost and radiation as well. The sensitivity and specificity of MRA in detecting PAD with stenosis > 50% is the same as CTA, 90% to 100% [43]. However, the procedure is time consuming, availability is limited, cost is high and there is a potential risk of gadolinium induced nephrogenic fibrosis in those with deranged kidney function.

## Strengths

Use of CT angiography as a non-invasive gold standard comparator is the greatest strength of our study and the first ever attempt to do so. CT angiography is highly diagnostic for PAD and is not operator dependent. Moreover, ABI and TBI recordings with a fully automated Doppler made the recording sessions convenient, and there was no need for interobserver comparison.

## Limitations

Subjects included in the study were selected from the largest tertiary care referral diabetes OPD of Eastern India. There might me some selection bias in terms of duration of diabetes. Most subjects in our study have duration of diabetes more than 13 years. A large scale multicentric study using similar modalities in a heterogenous population will be more informative and substantiated. Longer duration of diabetes is an independent risk factor for PAD. Secondly, the study included more male than female participants, though we selected subjects randomly who walked into our diabetes OPD.

## Conclusion

Ankle brachial index commonly used to screen for PAD fails to detect all cases particularly in presence of arterial wall calcification. Both a raised ABI (> 1.3) and a so-called normal ABI (0.9–1.3) may miss confirmed PAD cases. Toe brachial index or TBI can be a useful alternative in such circumstances because toe arteries are too small caliber to be affected by wall calcification. TBI has good sensitivity as a screening tool for PAD in T2D. ABI and TBI also have good correlation for a low ABI, but this correlation is low or absent for raised or so-called normal ABI. Our study is the first to compare ABI and TBI with CT angiography and prove TBI scores way ahead of ABI. The currently used cut-off of TBI (0.6) provides the best sensitivity and specificity for PAD detection but we propose 1.0 as the upper limit cut-off of ABI for best sensitivity and specificity as derived from ROC. We therefore recommend that TBI may replace ABI, however due to logistic reasons, ABI may continue to be used as an initial screening tool given its widespread availability and low cost. However, TBI should be the bedside tool of choice in detecting PAD in T2D with ABI > 0.9

### Supplementary Information

**Additional file 1: Table S1. **Correlation of ABI and TBI across various ranges of ABI.

## Data Availability

Included in the manuscript.

## Abbreviations

PAD: Peripheral arterial disease
ABI: Ankle brachial index
MAC: Medial arterial calcification
TBI: Toe brachial Index
OPD: Outpatient department
T2D: Type 2 diabetes
eGFR: Estimated glomerular filtration rate
LDL: Low density lipoprotein
BMI: Body mass index
HDL: High density lipoprotein
NPV: Negative predictive value
PPV: Positive predictive value
LR: Likelihood ratio
CVA: Cerebrovascular accident
CAD: Coronary artery disease
AUC: Area under the curve
ROC: Receiver operating curve

## References

[1] American Diabetes Association (2003). Peripheral arterial disease in people with diabetes. Diabetes Care.

[2] Criqui MH (2001). Peripheral arterial disease: epidemiological aspects. Vasc Med.

[3] Ugwu E, Anyanwu A, Olamoyegun M (2021). Ankle brachial index as a surrogate to vascular imaging in evaluation of peripheral artery disease in patients with type 2 diabetes. BMC Cardiovasc Disord.

[4] Rooke TW, Hirsch AT, Misra S (2011). 2011 ACCF/AHA Focused Update of the Guideline for the Management of Patients with Peripheral Artery Disease (updating the 2005 guideline): a report of the American College of Cardiology Foundation/American Heart Association Task Force on Practice Guidelines. J Am Coll Cardiol.

[5] Williams DT, Harding KG, Price P (2005). An evaluation of the efficacy of methods used in screening for lower-limb arterial disease in diabetes. Diabetes Care.

[6] Okamoto K, Oka M, Maesato K (2006). Peripheral arterial occlusive disease is more prevalent in patients with haemodialysis: comparison with the findings of multidetector-row computed tomography. Am J Kidney Dis.

[7] Aerden D, Massaad D, von Kemp K (2011). The ankle-brachial index and the diabetic foot: a troublesome marriage. Ann Vasc Surg.

[8] Stabley JN, Towler DA (2017). Arterial calcification in diabetes mellitus: preclinical models and translational implications. Arterioscler Thromb Vasc Biol.

[9] Hirsch AT, Haskal ZJ, Hertzer NR (2006). ACC/AHA Guidelines for the Management of Patients with Peripheral Arterial Disease (lower extremity, renal, mesenteric, and abdominal aortic): a collaborative report from the American Associations for Vascular Surgery/Society for Vascular Surgery, Society for Cardiovascular Angiography and Interventions, Society for Vascular Medicine and Biology, Society of Interventional Radiology, and the ACC/AHA Task Force on Practice Guidelines (writing committee to develop guidelines for the management of patients with peripheral arterial disease)—summary of recommendations. J Vasc Interv Radiol.

[10] Carter SA, Lezack JD (1971). Digital systolic pressures in the lower limb in arterial disease. Circulation.

[11] Ramsey DE, Manke DA, Sumner DS (1983). Toe blood pressure: a valuable adjunct to ankle pressure measurement for assessing peripheral arterial disease. J Cardiovasc Surg.

[12] Brooks B, Dean R, Patel S, Wu B, Molyneaux L, Yue DK (2001). TBI or not TBI: that is the question. Is it better to measure toe pressure than ankle pressure in diabetic patients?. Diabet Med.

[13] Second European Consensus: Document on chronic leg ischemia. Eur J Vasc Surg. 1992;6(Supp 1):1-32. 1533191

[14] TASC: Management of peripheral arterial disease: Transatlantic Intersociety Consensus. Eur J Vasc Endovasc Surg. 2000;19(Suppl. A): S1–S250 10957904

[15] Rooke TW, Hirsch AT, Misra S (2012). 2011 ACCF/AHA focused update of the guideline for the management of patients with peripheral artery disease (updating the 2005 guideline): a report of the American College of Cardiology Foundation/American Heart Association Task Force on Practice Guidelines, developed in collaboration with the Society for Cardiovascular Angiography and Interventions, Society of Interventional Radiology, Society for Vascular Medicine, and Society for Vascular Surgery. Catheter Cardiovasc Interv.

[16] US Preventive Services Task Force (2018). Screening for peripheral artery disease and cardiovascular disease risk assessment with the ankle-brachial index: US preventive services task force recommendation statement. JAMA.

[17] Park SC, Choi CY, Ha YI, Yang HE (2012). Utility of toe-brachial index for diagnosis of peripheral artery disease. Arch Plast Surg.

[18] Fleiss J (1986). Reliability of measurement.

[19] Landis JR, Koch GG (1977). The measurement of observer agreement for categorical data. Biometrics.

[20] Soyoye DO, Abiodun OO, Ikem RT, Kolawole BA, Akintomide AO (2021). Diabetes and peripheral artery disease: a review. World J Diabetes.

[21] Ikem R, Ikem I, Adebayo O, Soyoye D (2010). An assessment of peripheral vascular disease in patients with diabetic foot ulcer. Foot (Edinb).

[22] Vitalis A, Lip GY, Kay M, Vohra RK, Shantsila A (2017). Ethnic differences in the prevalence of peripheral arterial disease: a systematic review and meta-analysis. Expert Rev Cardiovasc Ther.

[23] Sahana P et al. High Prevalence of neuropathy and peripheral arterial disease in type 2 diabetes in a tertiary care centre in Eastern India. Int J Endocrinol. 2000, 2.

[24] Makkar BM, Sharma JK, Jaggi S, Soota K, Gupta V, Khurana G (2018). Low prevalence of peripheral arterial disease in Type 2 diabetes patients in North India. Diabetes.

[25] Krishnan MN, Geevar Z, Mohanan PP, Venugopal K, Devika S (2018). Prevalence of peripheral artery disease and risk factors in the elderly: a community based cross-sectional study from northern Kerala, India. Indian Heart J.

[26] Newman AB (2000). Peripheral arterial disease: insights from population studies of older adults. J Am Geriatr Soc.

[27] Schramm K, Rochon PJ (2018). Gender differences in peripheral vascular disease. Semin Intervent Radiol.

[28] Hamburg NM, Creager MA (2017). Pathophysiology of Intermittent Claudication in peripheral artery disease. Circ J.

[29] Zemaitis MR, Boll JM, Dreyer MA. Peripheral Arterial Disease. 2023 May 23. In: StatPearls [Internet]. Treasure Island (FL): StatPearls Publishing; 2024. 28613496

[30] Criqui MH, Matsushita K, Aboyans V, Hess CN, Hicks CW, Kwan TW, McDermott MM, Misra S, Ujueta F, American Heart Association Council on Epidemiology and Prevention, Council on Arteriosclerosis, Thrombosis and Vascular Biology, Council on Cardiovascular Radiology and Intervention, Council on Lifestyle and Cardiometabolic Health, Council on Peripheral Vascular Disease, Stroke Council (2021). Lower extremity peripheral artery disease: contemporary epidemiology, management gaps, and future directions: a scientific statement from the American Heart Association. Circulation.

[31] Hiatt WR, Jones DN (1992). The role of hemodynamics and duplex ultrasound in the diagnosis of peripheral arterial disease. Curr Opin Cardiol.

[32] Jelinek H, Austin M (2006). The ankle-brachial index in clinical decision making. Foot.

[33] Bhasin N, Scott DJ (2007). Ankle Brachial Pressure Index: identifying cardiovascular risk and improving diagnostic accuracy. J R Soc Med.

[34] Allen J, Overbeck K, Nath AF, Murray A, Stansby G (2008). A prospective comparison of bilateral photoplethysmography versus the ankle-brachial pressure index for detecting and quantifying lower limb peripheral arterial disease. J Vasc Surg.

[35] Abouhamda A, Alturkstani M, Jan Y (2019). Lower sensitivity of ankle-brachial index measurements among people suffering with diabetes-associated vascular disorders: a systematic review. SAGE Open Med.

[36] Stein R, Hriljac I, Halperin JL, Gustavson SM, Teodorescu V, Olin JW (2006). Limitation of the resting ankle-brachial index in symptomatic patients with peripheral arterial disease. Vasc Med.

[37] Álvaro-Afonso FJ, Lázaro-Martínez JL, Aragón-Sánchez J, García-Morales E, García-Álvarez Y, Molines-Barroso RJ (2015). What is the clinical utility of the ankle-brachial index in patients with diabetic foot ulcers and radiographic arterial calcification?. Int J Low Extrem Wounds.

[38] Tehan PE, Barwick AL, Sebastian M, Chuter VH (2017). Diagnostic accuracy of resting systolic toe pressure for diagnosis of peripheral arterial disease in people with and without diabetes: a cross-sectional retrospective case-control study. J Foot Ankle Res.

[39] Lehto S, Niskanen L, Suhonen M, Rönnemaa T, Laakso M (1996). Medial artery calcification. A neglected harbinger of cardiovascular complications in non-insulin-dependent diabetes mellitus. Arterioscler Thromb Vasc Biol.

[40] Neubauer B (1971). A quantitative study of peripheral arterial calcification and glucose tolerance in elderly diabetics and non-diabetics. Diabetologia.

[41] Young MJ, Adams JE, Anderson GF, Boulton AJ, Cavanagh PR (1993). Medial arterial calcification in the feet of diabetic patients and matched non-diabetic control subjects. Diabetologia.

[42] Catalano C, Fraioli F, Laghi A, Napoli A, Bezzi M, Pediconi F, Danti M, Nofroni I, Passariello R (2004). Infrarenal aortic and lower-extremity arterial disease: diagnostic performance of multi-detector row CT angiography. Radiology.

[43] Takahashi EA, Kinsman KA, Neidert NB, Young PM (2019). Guiding peripheral arterial disease management with magnetic resonance imaging. Vasa.

