# Correction: Toe brachial index and not ankle brachial index is appropriate in initial evaluation of peripheral arterial disease in type 2 diabetes

**DOI:** 10.1186/s13098-024-01309-9

**Published:** 2024-03-21

**Authors:** Pankaj Singhania, Tapas Chandra Das, Chiranjit Bose, Asif Mondal, Rana Bhattacharjee, Archana Singh, Satinath Mukhopadhyay, Subhankar Chowdhury

**Affiliations:** 1 Department of Endocrinology and Metabolism, Institute of Post Graduate Medical Education and Research, Kolkata, India; 2 Department of Radiodiagnosis, Institute of Post Graduate Medical Education and Research, Kolkata, India; 3 Department of Endocrinology, Medical College, Kolkata, India

**Correction: Diabetology & Metabolic Syndrome (2024)16: 52**

10.1186/s13098-024-01291-2

Following publication of the original article [1], the authors would like to correct the number 0.9 in the conclusion under the main heading of abstract.

The sentence currently reads:

## CONCLUSION

ABI < 0.9 detects PAD reliably, but presence of PAD in patients with ABI **> 9.0** including the normal of ABI (0.9–1.3) can be confirmed with TBI, which correlated strongly with CTA. TBI is also non-inferior for PAD detection, when ABI < 0.9. TBI and not ABI can be utilized for assessment of PAD in subjects with T2D.

The sentence should read:

## CONCLUSION

ABI < 0.9 detects PAD reliably, but presence of PAD in patients with ABI **>** 0.9 including the normal of ABI (0.9–1.3) can be confirmed with TBI, which correlated strongly with CTA. TBI is also non-inferior for PAD detection, when ABI < 0.9. TBI and not ABI can be utilized for assessment of PAD in subjects with T2D.
