# Supplementary material for: Toe brachial index and not ankle brachial index is appropriate in initial evaluation of peripheral arterial disease in type 2 diabetes
Source: Diabetol Metab Syndr. 2024 Feb 27;16:52. doi: 10.1186/s13098-024-01291-2 (PMC10898040; doi:10.1186/s13098-024-01291-2)
Supplement: Supplementary file 1 — Additional file 1: Table S1. Correlation of ABI and TBI across various ranges of ABI. [file 13098_2024_1291_MOESM1_ESM.docx]

**Table S1: Correlation of ABI and TBI across various ranges of ABI**

|  | **N** | **r value** | **p-value** |
| --- | --- | --- | --- |
| **ABI vs TBI** | **175** | **0.1875** | **0.0130** |
| **ABI ≤ 1.3 vs TBI** | **167** | **0.2066** | **0.0082** |
| **ABI >1.3 vs TBI** | 8 | **0.2623** | **0.4102** |
| **ABI 0.9-1.3 vs TBI** | **146** | **-0.07661** | **0.3580** |
| **ABI ≥ 0.9 vs TBI** | **154** | **-0.01934** | **0.8143** |
| **ABI < 0.9 vs TBI** | **21** | **0.7367** | **0.0001** |
